# Supplementary material for: Pre-screening workers to overcome bias amplification in online labour markets
Source: PLoS One. 2021 Mar 23;16(3):e0249051. doi: 10.1371/journal.pone.0249051 (PMC7987151; doi:10.1371/journal.pone.0249051)
Supplement: S1 Fig — (DOCX) [file pone.0249051.s001.docx]

**S1**
