# Supplementary figures and images for: Pre-screening workers to overcome bias amplification in online labour markets
Source: PLoS One. 2021 Mar 23;16(3):e0249051. doi: 10.1371/journal.pone.0249051 (PMC7987151; doi:10.1371/journal.pone.0249051)

**S2:**

**(A)**

**
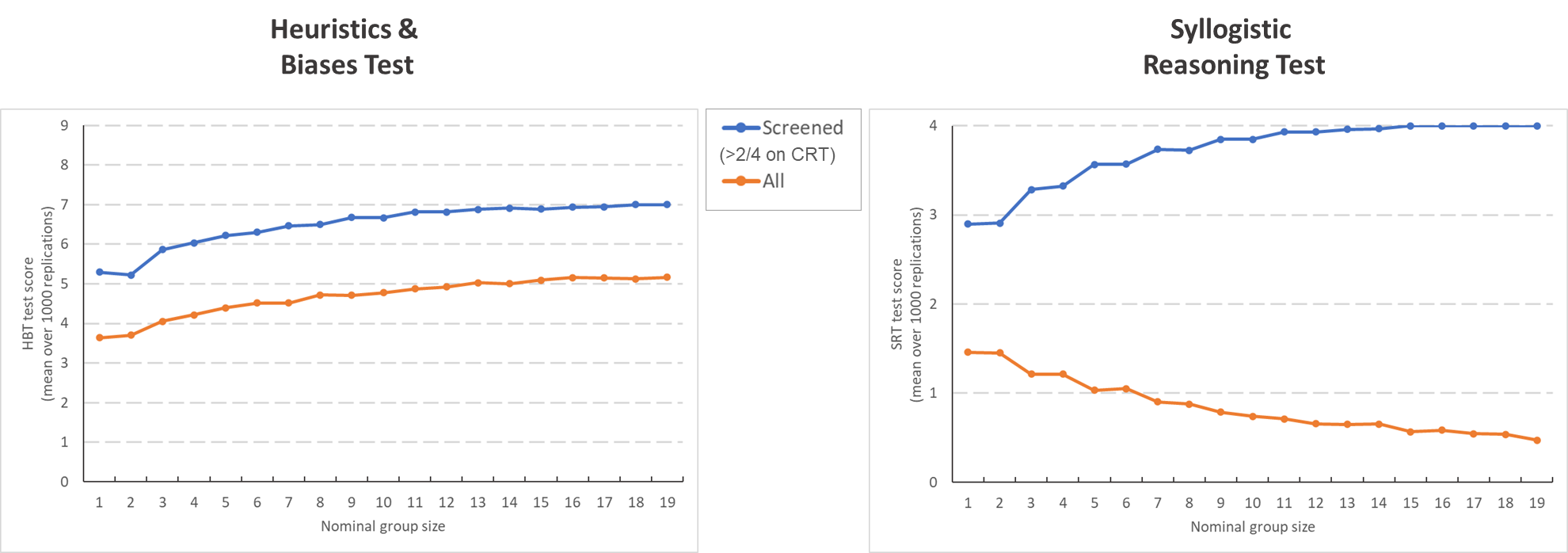
**

**(B)**

**
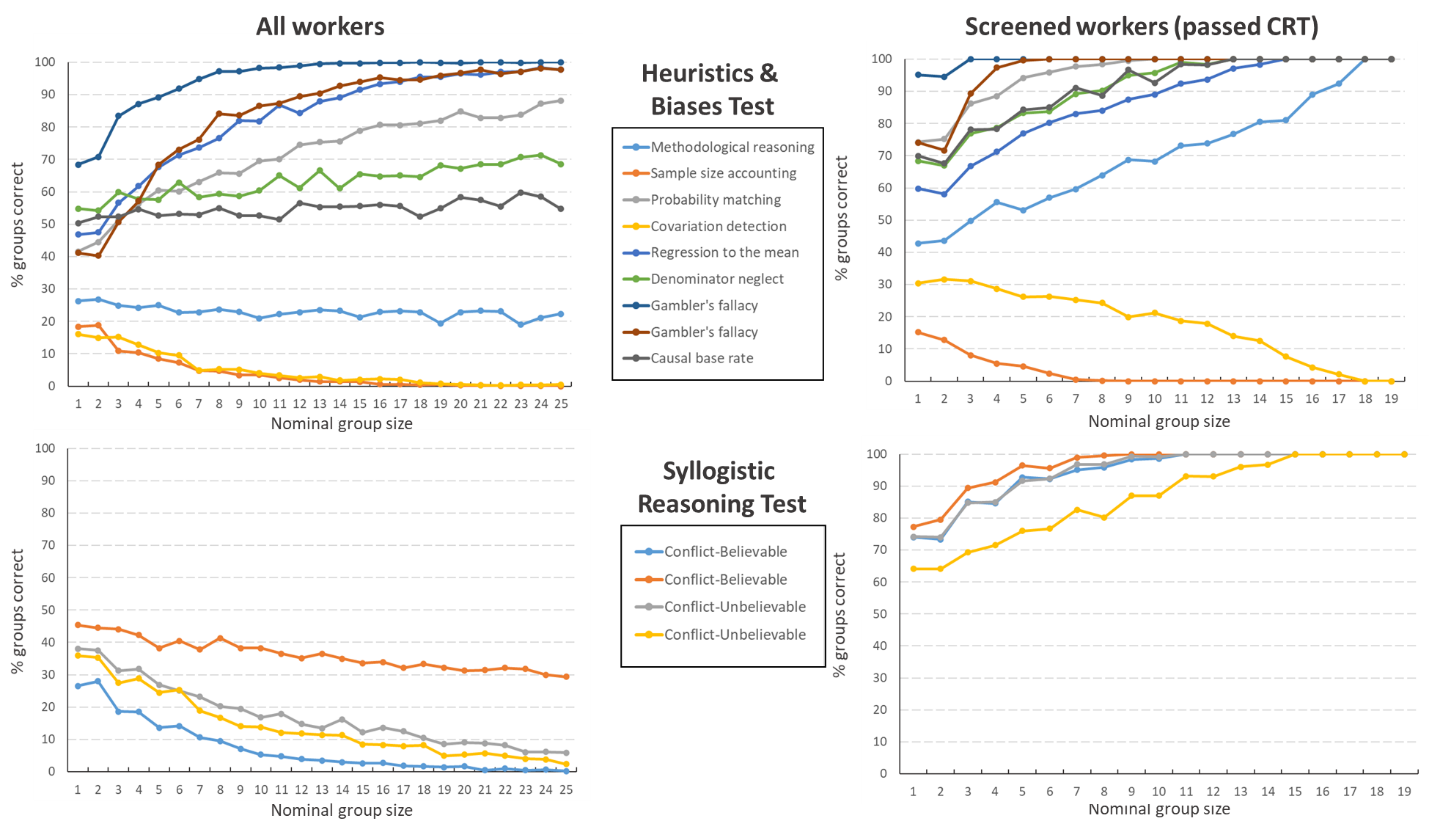
**

Supplement: S2 Fig — We screened N = 19 workers who answered at least 3 of the 4 CRT questions correctly and these workers were thus singled out for comparison against the full sample. As a result, the maximum nominal group size we could investigate was 19. Panel (A) shows the overall performance of nominal groups on the Heuristics and Biases Test and the Syllogistic Reasoning Test; panel (B) shows the item-by-item performance of nominal groups on the Heuristics and Biases Test and the Syllogistic Reasoning Test. (DOCX) [file pone.0249051.s002.docx]
