# Supplementary material for: Pre-screening workers to overcome bias amplification in online labour markets
Source: PLoS One. 2021 Mar 23;16(3):e0249051. doi: 10.1371/journal.pone.0249051 (PMC7987151; doi:10.1371/journal.pone.0249051)
Supplement: Attachment — Submitted filename: Vercammen_Response to reviewers.docx [file pone.0249051.s003.docx]

**RESPONSE TO REVIEWERS**

*We provide our point-by-point response to the reviewers’ comments in italics below.*

1. Is the manuscript technically sound, and do the data support the conclusions?

The manuscript must describe a technically sound piece of scientific research with data that supports the conclusions. Experiments must have been conducted rigorously, with appropriate controls, replication, and sample sizes. The conclusions must be drawn appropriately based on the data presented.

Reviewer #1: Yes

Reviewer #2: Yes

*Thank you. To answer Reviewer#2’s queries with regard to the external validity and causality of the findings (mentioned below), the revised manuscript now clearly outlines the limitations of the chosen design and the implications of this for future research [Lines 476-486 ]*

2. Has the statistical analysis been performed appropriately and rigorously?

Reviewer #1: Yes

Reviewer #2: Yes

*Thank you. No further changes have been made to the analyses.*

3. Have the authors made all data underlying the findings in their manuscript fully available?

The [PLOS Data policy](http://www.plosone.org/static/policies.action#sharing) requires authors to make all data underlying the findings described in their manuscript fully available without restriction, with rare exception (please refer to the Data Availability Statement in the manuscript PDF file). The data should be provided as part of the manuscript or its supporting information, or deposited to a public repository. For example, in addition to summary statistics, the data points behind means, medians and variance measures should be available. If there are restrictions on publicly sharing data—e.g. participant privacy or use of data from a third party—those must be specified.

Reviewer #1: Yes

Reviewer #2: No

*The full dataset and other relevant information such as the analysis code and the experimental materials are provided in full and referenced in the methods section [Line 132].*

4. Is the manuscript presented in an intelligible fashion and written in standard English?

PLOS ONE does not copyedit accepted manuscripts, so the language in submitted articles must be clear, correct, and unambiguous. Any typographical or grammatical errors should be corrected at revision, so please note any specific errors here.

Reviewer #1: Yes

Reviewer #2: Yes

*Thank you. We have ensured that our subsequent edits also meet the expected English language standard.*

5. Review Comments to the Author

Please use the space provided to explain your answers to the questions above. You may also include additional comments for the author, including concerns about dual publication, research ethics, or publication ethics. (Please upload your review as an attachment if it exceeds 20,000 characters)

Reviewer #1: Hello, Its honor for me to review your research manuscript . Please improve the result & discussion part . This part need improvement further and also improve the conclusion part . I hope you will improve it

*The reviewer did not specify which aspects of the results, discussion or conclusion required polishing. However, based on this advice and on the questions raised by the second reviewer, we have made revisions to highlight the major limitations of the work, explore future research opportunities, and identify potential real-life implications of our findings, without extrapolating beyond the bounds of what the results indicate. We hope this addresses Reviewer#1’s queries and satisfies Reviewer2.*

Reviewer #2: Comments

1. The revised introduction should state the contribution of the paper to the earlier literature.

*We have clarified that our paper provides empirical verification based on theoretical assumptions about the occurrence of reasoning errors in individuals and the effects of aggregation in crowdsourcing/crowdsolving. We point out that these assumptions have not yet been tested specifically in a crowdsolving setting where randomly selected individual members of an online panel provide independent results that are aggregated post-hoc. While it can be assumed that cognitive biases are just as prevalent in these types of samples and situations, this is an untested assumption, and, more importantly, we also do not know how group size affects collective performance when erroneous thinking/responding is common in individuals. We have made this point clearer in the introduction. The second unresolved issue that we address here is whether a simple strategy that has been demonstrated to effectively identify “effortful thinkers” could be applied to mitigate the effects of cognitive bias in crowdsolving. We now emphasise these specific novel aspects of our paper more clearly in the revised manuscript [Lines 98-110].*

2. The sample size is relatively small (page 6). What is the external validity of the findings that are presented in the paper?

*We agree with the reviewer that this is an important issue. However, compared to other papers (e.g. the seminal paper on crowdsourced collective intelligence by Kosinski et al 2012), our study has a larger base sample. To ensure reliability of our sample size analysis, we repeated the sub-sampling procedure and test score calculation 1000 times for each group size n. Of course, we cannot claim that the 105 individuals that formed our base sample are entirely representative of “online workers”. However, we did not specify any restrictions on the selection of individuals by the Qualtrics panel service, and thus accessed a pool of participants that are broadly representative of casual online labourers. We reflect on the implications of this approach in the revision [Lines 435-439].*

3. The description of the data should be improved. Was non-response to the survey random or not? This information would be useful in order to better understand the estimation results that are presented in the paper.

*Because of the way these online labour markets work, the requester does not have access to information about non-responses. In short, we cannot provide data on whether non-response was random or not. It would be interesting, from a theoretical perspective, to understand how this form of sampling bias might have affected nominal group performance. However, from a practical point of view, our approach reflects the reality of online labour markets. This consideration also speaks to the issue of our base sample’s representativeness, which we have discussed above. We expand on these issues in our limitations section [Lines 435-443].*

4. Do the data contain (survey) weights? Are they used in the estimations or not?

*No, the survey data was not weighted in our analyses. We now specify this in the methods section [Line 138]. Of course, one could view excluding respondents on the basis of the screening test as an extreme form of weighting (i.e. survey weight = 0 if they did not pass the screening test). Logically, one other approach could be to assign a weight to individual responses on the basis of some indicator variable (e.g. score on the screening test, education level or another way of classifying reasoning ability). However, our aim was to examine baseline nominal group performance (i.e. without preselection or intervention) and then to test a simple and cost-effective strategy for quickly “weeding out” responses that would unduly and negatively affect nominal group performance. Weighting is a more resource intensive and – from a task requester perspective – costly option to achieve this goal. It was beyond the scope of the current investigation to address this.*

*We note that we provide a full description and a copy of the raw data, and it is therefore open to any further analyses and exploration by interested parties.*

5. The statistical analyses do not address causal questions. Therefore, the paper reports (conditional) correlations between the variables of interest using cross-sectional variation. But the measures that are used in the empirical specifications are subjective. This implies that the unobserved individual-level characteristics such as personality traits may have a substantial influence on all variables that are used in the analysis. This limits the conclusions that can be drawn from the estimates.

*We agree with the reviewer that the nature of the study does not allow us to draw causal links, and that there are traits or characteristics that we did not measure that might affect the performance of online workers. However, from a practical perspective causality is not a prerequisite. That is, we demonstrate that using the CRT to screen workers could be a simple and easy tool to mitigate against the negative influence of biased reasoning in a general sample of online workers. Task requesters will rarely have a detailed understanding of the characteristics of their online workforce. More elaborate subsampling on the basis of education, personality traits etc. would be more arduous and costly than using the CRT, which provides a shortcut to optimising performance that is reasonable, simple to implement and does not require the worker to divulge more sensitive or personal information (e.g. about their personality, their educational history, their occupation).*

*Nevertheless, we agree that as we have tested the applicability of this approach on a small set of reasoning tasks, the broader use of such a shortcut remains to be demonstrated [Lines 468-473]. Indeed, in other types of challenges, some of the variables mentioned by the reviewer may be more important or may play a moderating role. We have included this consideration in our revised limitations section [Lines 444-448 ]*

6. The concluding section could discuss more about the practical lessons that stem from the results.

*We thank the reviewer for this suggestion and have made the requested edits, considering the findings from the perspective of a task requester using online labour markets [Lines 448-455].*
